# Managing Hypertension in Older Adults

**DOI:** 10.1007/s11906-023-01289-7

**Published:** 2023-12-27

**Authors:** Brent M. Egan, Holly J. Mattix-Kramer, Jan N. Basile, Susan E. Sutherland

**Affiliations:** 1 American Medical Association, Improving Health Outcomes, 2 West Washington Street, Suite 601, Greenville, SC 29601 USA; 2 Department of Public Health Sciences and Medicine, Loyola University Chicago Loyola University Medical Center, Maywood, IL USA; 3 Department of Medicine, Division of Cardiology, Medical University of South Carolina, Charleston, SC USA

**Keywords:** Hypertension, Isolated systolic hypertension, Older adults, Antihypertensive medications, Lifestyle change, Cardiovascular events

## Abstract

**Purpose of Review:**

The population of older adults 60–79 years globally is projected to double from 800 million to 1.6 billion between 2015 and 2050, while adults ≥ 80 years were forecast to more than triple from 125 to 430 million. The risk for cardiovascular events doubles with each decade of aging and each 20 mmHg increase of systolic blood pressure. Thus, successful management of hypertension in older adults is critical in mitigating the projected global health and economic burden of cardiovascular disease.

**Recent Findings:**

Women live longer than men, yet with aging systolic blood pressure and prevalent hypertension increase more, and hypertension control decreases more than in men, i.e., hypertension in older adults is disproportionately a women’s health issue. Among older adults who are healthy to mildly frail, the absolute benefit of hypertension control, including more intensive control, on cardiovascular events is greater in adults ≥ 80 than 60–79 years old. The absolute rate of serious adverse events during antihypertensive therapy is greater in adults ≥ 80 years older than 60–79 years, yet the excess adverse event rate with intensive versus standard care is only moderately increased. Among adults ≥ 80 years, benefits of more intensive therapy appear non-existent to reversed with moderate to marked frailty and when cognitive function is less than roughly the twenty-fifth percentile. Accordingly, assessment of functional and cognitive status is important in setting blood pressure targets in older adults.

**Summary:**

Given substantial absolute cardiovascular benefits of more intensive antihypertensive therapy in independent-living older adults, this group merits shared-decision making for hypertension targets.

**Supplementary Information:**

The online version contains supplementary material available at 10.1007/s11906-023-01289-7.

## Introduction

The population of older adults is growing faster than the general population globally and prevalent hypertension and cardiovascular risk also rise sharply with aging [1–4]. Thus, the health and economic burden of hypertension and related complications will likely grow faster than the global population and economy. These factors collectively magnify the value of highly scalable, cost-effective management of hypertension in older adults.

This review on managing hypertension is older adults is provided to assist healthcare providers, public health officials and policy makers in their efforts to mitigate the burden of uncontrolled hypertension and the health and economic toll of related complications. The review addresses eight key items including: (i) the current and projected numbers of older adults with hypertension (ii) the absolute risk for major adverse cardiovascular events in older adults (iii) the estimated number-needed-to treat (NNT) for benefit and number-needed-to-harm (NNH) (iv) women’s health perspective on hypertension in older adults (v) the importance of assessing comorbid chronic conditions as well as physical and cognitive status in setting treatment targets and when selecting a treatment plan (vi) lifestyle changes in older adults with hypertension (vii) pharmacotherapy of hypertension in older adults, while balancing benefit and risk of treatment and challenging the notion that start low and go slow is best for most older adults (viii) periodic reassessment of comorbid conditions, physical and cognitive status, and hypertension target.

### Increase in Numbers of Older Adults and Impact On the Global Burden of Hypertension and Cardiovascular Disease

**Fig. 1:**
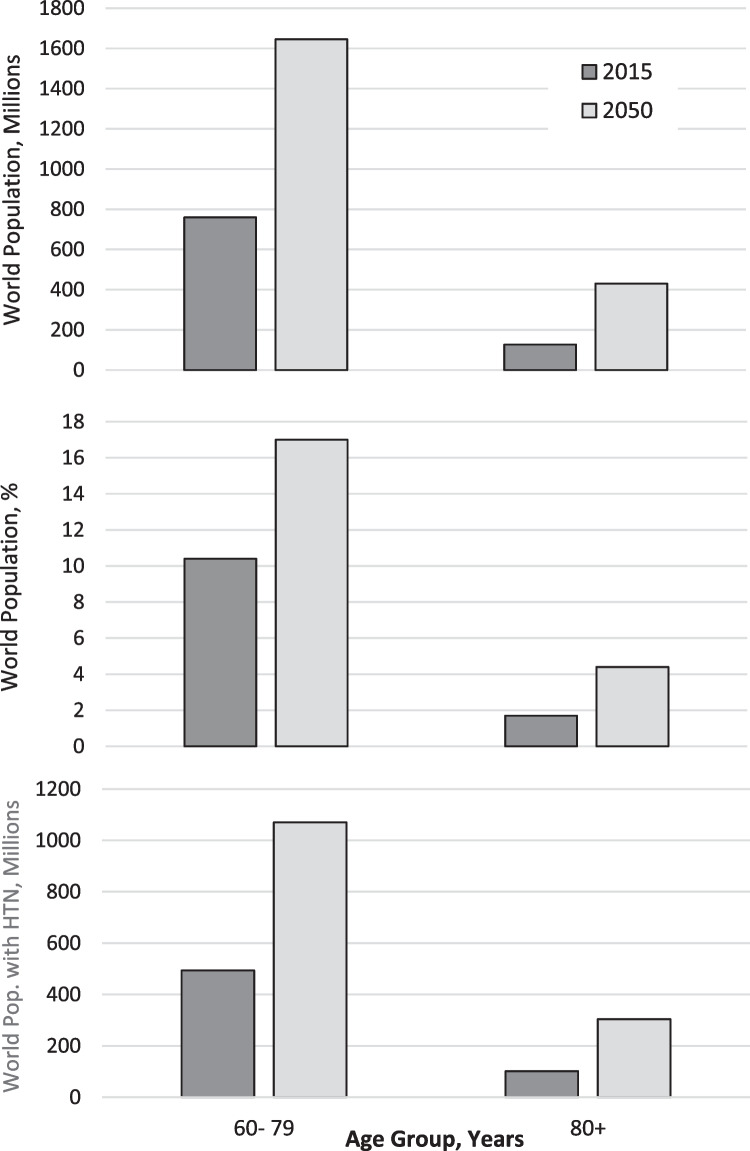
The numbers and percentages of adults 60–79 and ≥ 80 years globally in 2015 and 2050. Legend. The global numbers of adults 60–79 and ≥80 years in 2015 and 2050 (top panel), respective percentages of the total global population (middle panel), and numbers with hypertension (bottom panel) are shown. The projected increases are large and have important implications for the global health and economic burden of hypertension and related cardiovascular disease

The number of adults 60–79 years is estimated to rise from 760 million in 2015 to 1,646 million in 2050 or from 10.4% to 17.0% of the world’s population (Fig. 1, top) [1]. The number of adults ≥ 80 years is projected to grow from 126.6 million in 2015 to 430.3 million in 2050 or from 1.7% to 4.4% of the world’s population (Fig. 1, middle).

#### Increase in Numbers of Older Adults with Hypertension

If we estimate that ~65% of adults 60–79 years and 80% of adults ≥ 80 years have hypertension defined by ≥ 140/ ≥ 90 mmHg or pharmacotherapy for hypertension, then the number of adults 60–79 years with hypertension would rise from roughly 494 million in 2015 to 1.07 billion in 2050. Concurrently, the number of adults ≥ 80 years with hypertension could rise from 101 million in 2015 to 344 million in 2050 (Fig. 1, bottom). In so, the number of older adults with hypertension in 2050 would exceed the total number of adults 30–79 years with hypertension globally in 2010 [5].

### Absolute Risk for Major Cardiovascular Events

Death from ischemic heart disease and stroke approximately double each decade from 40–49 through 80–89 years [3]. The risk of fatal ischemic heart disease and stroke double for each 20 mmHg increase in systolic BP above 115 mmHg [3]. To provide a rough estimate of actual numbers, data from the placebo group in the Hypertension and Very Elderly Trial (HYVET) was used. Among adults ≥ 80 years with hypertension in HYVET randomized to placebo, ~ 5% had a major cardiovascular disease event (CVDE) yearly. Since HYVET enrolled adults with systolic BP ≥ 160 mmHg, which is more severe than the average adult of that age group, an annual CVDE rate of 4% rather than 5% was used for estimating future incidence. Adults with hypertension 70–79 years old were assigned a 2% annual CVDE rate and those 60–69 years a 1% annual rate. An annual CVDE rate of 1.5% was assigned to adults 60–79 years old, recognizing more adults are 60–69 than 70–79 years.

**Fig. 2:**
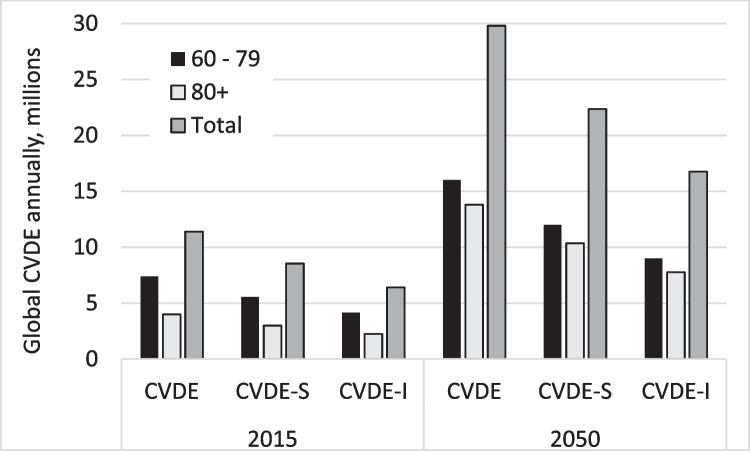
Projected number of cardiovascular disease events in 2015 and 2050 in older adults with and without antihypertensive therapy. Abbreviations: CVDE, cardiovascular disease events; CVDE-S, estimated number of CVDE with standard antihypertensive therapy; CVDE-I, estimated number of CVDE with intensive antihypertensive therapy

Given these assumptions, CVDE would more than double from 7.4 to 16 million from 2015 to 2050 absent risk reducing interventions (Fig. 2). CVDE in adults ≥ 80 years would rise from 4 million in 2015 to 13.8 million in 2050.

### Estimated Benefit of Antihypertensive Therapy for Reducing CVDE

Numbers-needed-to-treat (NNT) for benefit on the primary outcomes were estimated for HYVET, SPRINT and, STEP and subdivided by age < 80 and ≥ 80 years (Table 1). To facilitate comparisons across studies, benefit (NNT) was estimated at 3.6 years, which was the median follow-up time in the SPRINT report on adults ≥ 80 years. In general, absolute benefit was greater (smaller NNT) in adults ≥ 80 years than < 80 years and with greater reductions in BP.

**Table 1:** Treating hypertension in older adults: Selected summary with focus on NNT to prevent primary outcome

**STEP 60– ≤ 80 years and SPRINT 50 − < 80 years**						
**Study**	**Design**	**Intervention**	**Control**	**In-study SBP** **Group Difference**	**Primary Outcome**	^**b**^**Primary Outcome** **NNT at ~ 3.6 years**
**STEP** [10••] **60–80 years**	Prospective Randomized	SBP 110 − < 130 Intensive	SBP 130 − < 150 Standard	135.9 vs 126.7 9.2 mmHg over intervention	Stroke, ACS, MI, CHF, A-fib, CV death	86 vs. standard Rx
^**a**^**SPRINT** [7, 9•] **50–79 years**	Randomized open-label	SBP < 120 Intensive	SBP 135 − < 140 Standard	134.5 vs. 121.1 13.4 mmHg over intervention	MI, ACS, stroke, CHF, CV death	45 vs. standard Rx

**HYVET and SPRINT 80 years and older**						
**Study**	**Design**	**Intervention**	**Control**	**In-study SBP** **Group Difference**	**Primary Outcome**	**Primary Outcome** **NNT at ~ 3.6 years**
**HYVET** [6]	Randomized, double-blind, placebo-control	BP target 150/80	BP ≤ 220/ ≤ 110 (upper limit)	158.5 vs 143.5 Δ 15 mmHg at 2 years	Fatal, non-fatal stroke	36 vs. placebo
**SPRINT** [9•]	Randomized to BP target (open-label treatment)	SBP target < 120 intensive	SBP target vs 135–139 standard	135.3 vs 123.9 Δ 11.5 mmHg over intervention	MI, ACS, stroke, CHF, CV death	20 vs. standard Rx
**SPRINT** [9•] **(MoCA)**	MOCA ≥ vs. < ~ 25 percentile	SBP target < 120 intensive	SBP target 135– 139 standard	135.3 vs 123.9 Δ 11.5 mmHg over intervention	MI, ACS, stroke, CHF, CV death	13 vs. standard Rx

^a^SPRINT data for 50–79 years obtained by subtracting results for adults ≥80 years from entire cohort for both treatment groups

^b^Primary outcome reported at 3.6 years for SPRINT participants 80 years and older. The outcome at 3.6 years was estimated across studies reported in this table

#### Comparison of Standard and Intensive Treatment Goals and Major Guideline Recommendations [6–8, 9•, 10••, 11–13••]

For this discussion, the goal for standard therapy is systolic BP < 140 and for intensive therapy < 130 mmHg. These targets, while not identical to stated goals in the original reports, reflect mean in-study systolic BP values in SPRINT and STEP. Based largely on SPRINT [7, 8], the 2017 ACC/AHA Guideline strongly recommended a systolic BP target < 130 for adults ≥ 65 years with no upper age limit [12].

In contrast, for adults 65–79 years without isolated systolic hypertension (ISH), the 2023 European Society of Hypertension (ESH) Guideline specified a target systolic BP 130–139, noting < 130 could be considered if treatment were well tolerated (Class I) [13••]. For adults 65–79 years with ISH, ESH recommended a primary systolic BP target 140–150 (Class I) with cautious consideration of 130–139 if treatment were well tolerated (Class I). For adults ≥ 80 years, the ESH recommended a target for office systolic BP 140–150 (Class I), noting a systolic BP target 130–139 may be considered if well tolerated but cautiously if diastolic BP is < 70 (Class II).

Assuming a 25% reduction in CVDE with standard therapy and an additional 25% reduction with intensive therapy [6–8, 9•, 10••], CVDE among adults 60–79 years in 2015 could have declined from 7.4 to 5.6 and 4.2 million, respectively (Fig. 2). In 2050, CVDEs would fall from 16 to 12 and 9 million with standard and intensive treatment, respectively. Among adults ≥ 80 years, CVDE are estimated at 4 million in 2015 and 13.8 million in 2050. Standard and intensive therapy would reduce these numbers to 3 and 2.3 million in 2015 and to 10.4 and 7.8 million in 2050, respectively. In both older age groups combined, nearly 30 million CVDE could occur in 2050 without intervention, declining to roughly 22 million and 17 million with standard and intensive therapy, respectively. Thus, millions of CVDE could be prevented.

Recognizing the multiple limitations of the projected reductions in CVDE with antihypertensive therapy is important. First, the evidence for benefit of antihypertensive treatment from randomized controlled trials in adults ≥ 80 years is very limited [6, 9•]. HYVET enrolled 3845 participants [6], and SPRINT included 1167 participants ≥ 80 years [9•]. Second, not all older adults are candidates for intensive therapy, especially those ≥ 80 years [11]. Clinical trials enrolled older adults who were living independently and free from health problems that significantly limited expected lifespan [6–8, 9•, 10••, 11–13••]. Study subjects did not have major mental or physical limitations or clinically significant orthostatic hypotension. Third, not all adults attain standard or intensive therapy goals when indicated. Fourth, there are risks from intensive antihypertensive therapy, quantifiable as number-needed-to-harm (NNH).

#### NNH With Intensive vs. Standard Treatment Goals

The number needed-to-treat (NNT) for benefit and NNH are important in assessing the benefit-to-risk ratio of active treatment vs. inaction or more vs. less intensive treatment. Unfortunately, data on serious adverse events (SAEs) in the published studies on older adults with hypertension are limited and permit only crude estimates of NNH.

#### HYVET

In the HYVET placebo group, 448 serious SAEs were reported vs. 358 with active treatment, although only three and two events, respectively, were attributed to the intervention [6]. In HYVET, active treatment was protective against SAEs.

#### SPRINT

SAEs only included hypotension, syncope, bradycardia, electrolyte abnormalities, and acute kidney injury or acute renal failure [7–9•]. SAEs are summarized in Table 2 for all subjects in SPRINT, those ≥ 80 years, for the subset of ≥ 80 years with scores on the Montreal Cognitive Assessment (MoCA) scores above approximately the twenty-fifth percentile, and for adults < 80 years.

**Table 2:** SPRINT estimates of number-needed-to-harm based on reported SAEs^a^

Rx Group	All (9361) / Events	≥ 80 years (1167) / Events	≥ 80 years (MoCA+ [754]^b^	< 80 years (8194)
Intensive	15.1% (707/ 4678)	34.1% (200/ 586)	32.3% (122/ 378)	12.4% (507/ 4092)
Standard	11.1% (519/ 4683)	28.6% (166/ 581)	26.9% (101/ 376)	8.6% (353/ 4102)
NNH	25	18	19	27

^a^SAEs included hypotension, syncope, bradycardia, electrolyte abnormality, injurious fall, or AKI or ARF) over the course of the trial. Estimated cumulative incidence of SAEs over the Intervention Period, comprised of hypotension, syncope, bradycardia, electrolyte abnormality, injurious fall, and AKI or ARF. Study participants may be counted in more than one SAE

^b^MoCA+ , Montreal Cognitive Assessment scores > 18 for participants with less than high school (HS) education and > 20 for ≥ HS

If we assume that an individual had only one SAE, then 15.1% of intensive and 11.1% of standard treatment participants ≥ 80 years old had an SAE during the SPRINT study [9•]. With an absolute difference of 4.0%, the NNH with intensive treatment is 25 over a median follow up of 3.73 years [7]. For all individuals ≥ 80 years, the absolute difference in SAEs was 5.5%, NNH 18 vs. NNT 20. For individuals ≥ 80 years with MoCA scores ≥ 25th percentile, the absolute difference between intensive and standard treatment was 5.3%, NNH 19 vs. NNT 20. And, for SPRINT participants 50–79, the absolute difference was 3.8%, NNH 27 vs. NNT 36. Of note, SAEs in SPRINT participants 50–79 years were not published separately but were estimated by subtracting SAEs in adults ≥ 80 years from the total population [7, 9•].

The ratio of NNT/NNH may serve as a crude estimate of benefit-to-risk, where lower ratios are more favorable. Based on SPRINT NNT and NNH data (Tables 1 and 2) [7, 9•], the ratios for intensive antihypertensive treatment in relatively healthy adults ≥80 years (NNT 20/NNH 18 all; NNT 13/NNH 19 MOCA ≥25^th^ percentile) were lower than for 50–79 years (NNT 45/NNH 19). However, it is important to note that SPRINT participants designated as frail had mild-moderate and not severe frailty [8, 11, 14, 15].

### Hypertension in Older Adults: An Under-recognized Health Equity Issue for Women

Women comprise 55 percent of the worldwide population ≥ 65 years and 62 percent of those ≥ 80 years [16]. Moreover, women show steeper increases than men in systolic BP and prevalent hypertension with advancing age, whereas hypertension control falls more [17, 18]. Thus, hypertension in older adults disproportionately impacts women.

#### Women, Aging, and BP

**Fig. 3:**
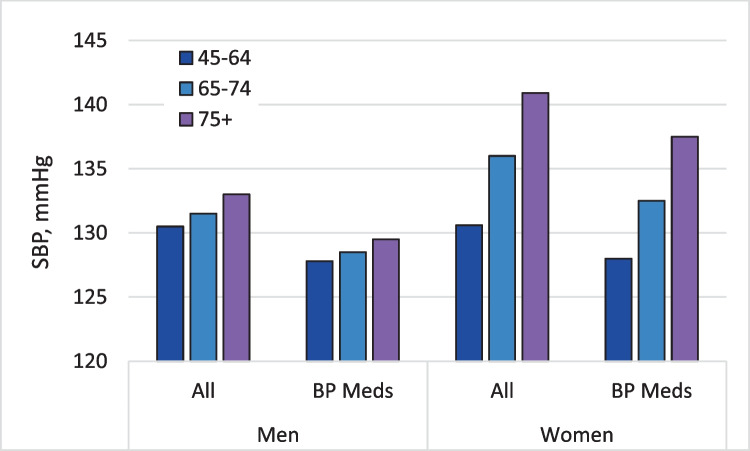
Systolic BP in adults by age group in men and women [17]. Legend. In the MESA longitudinal cohort study, systolic BP increased more with aging in women than men for all adults and only for those on BP meds (antihypertensive medications). Thus, the prevalence of hypertension increases more with age in women than men and control rates fall more than in men

Systolic BP increases more with age in women than men based on longitudinal data from the Multi-Ethnic Study of Atherosclerosis (MESA [Fig. 3]) [17]. Men and women in MESA had similar systolic BP at ages 45–64. Systolic BP increased ~ 10 mmHg in women from ages 45–64 to ≥ 75 years, whereas systolic BP rose ~ 2.5 mmHg in men over this age range. In MESA, diastolic BP from ages 45–64 to 75 declined roughly 6 mmHg in men and 2 mmHg in women. Thus, pulse pressure, another cardiovascular risk factor [19], increased more with age in women than men at roughly 12 vs 8.5 mmHg.

The greater age-related increase in systolic BP and pulse pressure in women than men presumably reflect differential changes in arterial stiffness [8, 19]. While declining estrogen with menopause may contribute to arterial stiffness with aging in women, randomization to estrogen replacement did not reduce systolic BP among post-menopausal participants in the Women’s Health Study [17]. However, pharmacological replacement of estrogen may not replicate naturally occurring estrogen [8, 20]. In addition to the potential role of diminished estrogen, women have significantly higher central BP relative to peripheral BP values than men at younger ages [20]. Sex difference in central BP is largely explained by shorter stature of women with more rapid return of reflected waves during systole in women, which augment central systolic BP [11, 20].

Height declines more with age in women than men [21]. By age 80, women lose an average of eight cm or three inches versus five cm or two inches in men. While height declines with age, the aortic arch and infrarenal aorta lengthen [22], which contributes to aortic tortuosity. A greater decline in height with age in women than men is consistent with evidence that aortic tortuosity is associated with female sex in addition to age and hypertension [23]. Yet, research on arterial tortuosity, the timing of reflected waves, and effects on systolic, diastolic, and pulse pressures remains limited.

Greater use of statins in men than women, especially before the menopause [24], may contribute to sex differences in age-related arterial stiffening [24]. Statins have only a small effect on BP [25] but slow progression of arterial stiffness with aging [26]. Thus, lesser use of statins in women than men, especially in mid-life, could contribute to greater arterial stiffening with age in women. Unisex risk calculators under-estimate the CVD risk of brachial BP in women relative to men [20]. Correcting this bias could potentially increase statin use in pre-menopausal women and mitigate their greater age-related arterial stiffening.

#### Benefits of Antihypertensive Therapy, Including Intensive Antihypertensive Therapy, in Older Women

HYVET, STEP, and the two SPRINT papers on older adults did not assess outcomes separately in women and men [6, 8, 9•, 10••]. In the primary SPRINT outcomes report [7], the hazard ratio for the primary outcome with intensive vs. standard treatment was statistically significant in men (0.72 [0.59–0.88)]) but not women (0.84 [0.62–1.14]). However, the hazard ratios in men and women were not significantly different.

### Importance of Comorbid Chronic Conditions and Physical and Cognitive Status in Setting Treatment Targets and Selecting a Treatment Plan in Older Adults with Hypertension

The presence of multiple chronic conditions rises sharply as a function of age and affects most older adults [27]. Most chronic conditions have indications for specific medication, leading to polypharmacy in many older adults, which, in turn increases drug-drug interactions, and the probability that compelling indications for one chronic condition will be contra-indicated for a concomitant condition, i.e., drug-disease interactions. Arterial stiffness, age-related autonomic changes, and polypharmacy raise risk for orthostatic hypotension. Advanced physical frailty and cognitive decline appear to neutralize the beneficial effects of more intensive BP control [9•, 11].

The evaluation should consider secondary causes of hypertension. While details exceed the scope of this review, common contributors to secondary hypertension in older adults include chronic kidney disease, sleep apnea, primary aldosteronism, hypothyroidism, and renal artery stenosis.

**Fig. 4:**
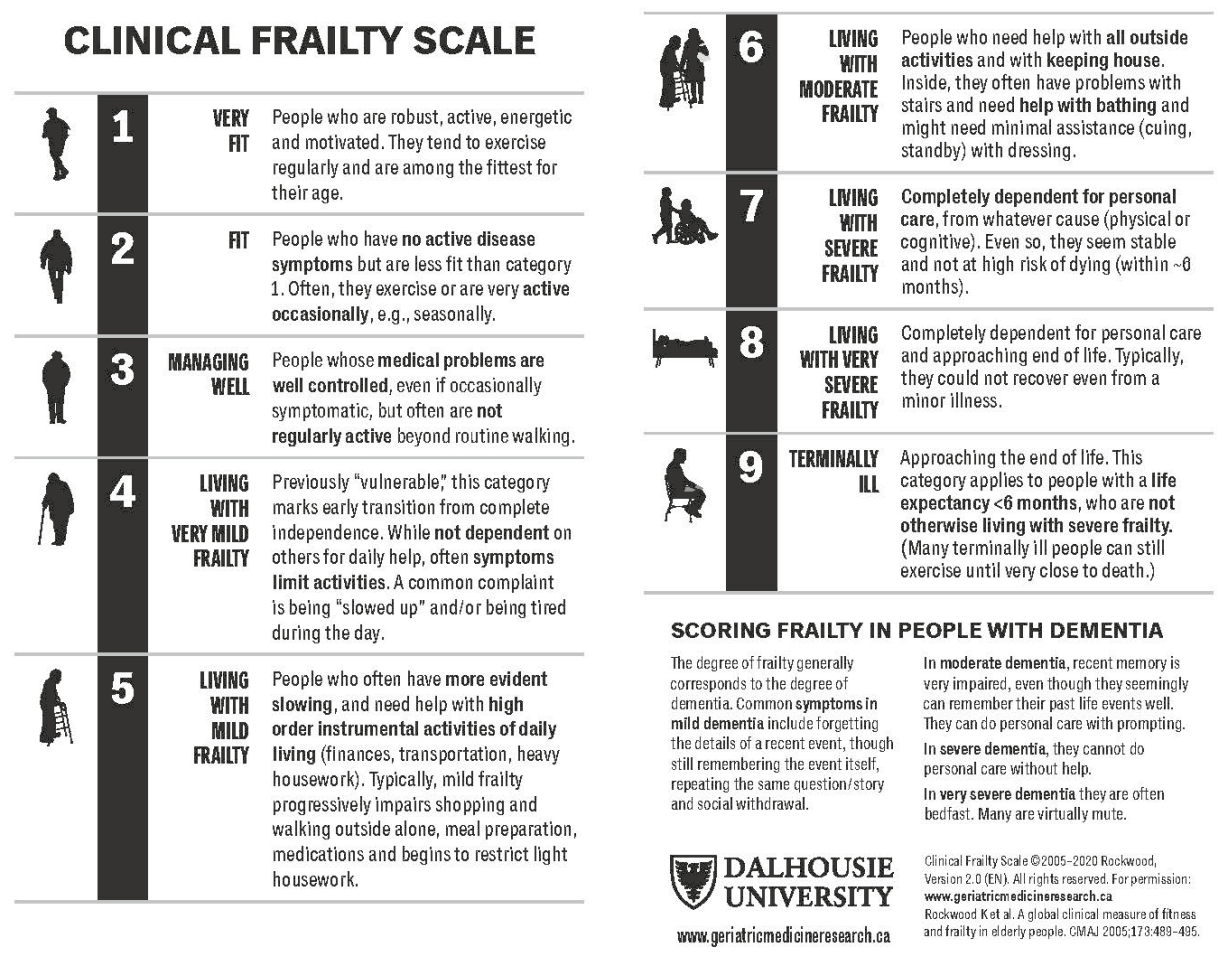
Provides guidance for assessing levels of frailty. Randomized trials of antihypertensive therapy have typically excluded individuals with moderate and severe frailty. Legend. A global clinical measure of fitness and frailty in elderly people. The clinical frailty scale defines nine levels of fitness and frailty with key features of each level provided above [14]

Quality of life is important, especially for older adults [28]. Thus, a comprehensive history, including medications, thorough physical examination, including a formal cognitive assessment, are relevant in older adults. Selected recommendations and tools for assessing physical and cognitive status and life priorities are available in citations and Fig. 4 [11, 14, 15]. This information is essential to developing an effective, patient-centered approach to setting hypertension treatment goals and selecting appropriate pharmacotherapy for older adults with hypertension.

### Lifestyle Changes in Older Adults with Hypertension

#### Overview

Several lifestyle interventions, described below, are effective for lowering BP in older adults. Lifestyle options for BP reduction in older adults include dietary changes without weight loss, weight loss, and physical activity. Caution is advised with weight loss interventions in older individuals as significant reduction of muscle mass, strength, and bone mineral density can occur [29].

#### Observational Study

The Healthy Ageing Longitudinal study in Europe (HALE) reported the effects of lifestyle factors on 10-year mortality in 2339 healthy men and women 70–90 years old in 11 European countries [30]. Compared to adults with 0–1 of four healthy lifestyle factors (Mediterranean Diet, physical activity, smoking, alcohol intake; 11% of sample), adults with three healthy factors (41% of sample) had less than half the multivariable adjusted hazard ratio for all-cause, coronary heart disease, cardiovascular disease, cancer and other-cause mortality.

#### Sodium Restriction

Salt-sensitivity is associated with age-related increases of BP, and salt-sensitivity increases with age [31]. In the randomized Trial of Nonpharmacologic Intervention in the Elderly (TONE) [32], 975 independently living adults 60–80 years without serious physical or mental illness were enrolled. Participants had BP < 145/ < 85 on antihypertensive monotherapy or single-pill combination including a diuretic and non-diuretic drug class. In TONE, a 40 mmol/d reduction in sodium, from ~ 3.5 to 2.5 g daily, lowered BP 4/2 mmHg [33]. The primary outcome of systolic BP ≥ 150 mmHg, diastolic BP ≥ 90 off medication, which was stopped at 3 months, restarting antihypertensive medication, or a cardiovascular event was reduced 42% (relative hazard ratio 0.68, p < 0.001) among adults randomized to reduced sodium [31]. The primary outcome also occurred less often in adults of African descent (hazard ratio 0.56, P = 0.005). The 2023 ESH Guideline did not recommend salt restriction for adults ≥ 80 years unless intake was > 10 g daily, or roughly > 170 mmol, due to concerns over nutritional status.

#### Mediterranean-style Diet

is moderate in sodium (< 170 mmol) and adherence to this diet can lower systolic BP 5.5 mmHg and decreases arterial stiffness in adults 65–79 years old after one year [34]. In this study, the Mediterranean diet appeared to be more effective for lowering BP in men than women. While the study included adults 60–79 years old, the Mediterranean diet likely has a favorable risk-to-benefit ratio in adults ≥ 80 years old as well.

#### Weight Loss

Among obese adults in TONE (n = 585, mean age 66 years), the relative hazard ratio for the primary outcome was reduced 30% with ~ 4 kg weight loss, 40% with sodium restriction, and 53% for weight loss and sodium restriction combined [32]. The authors concluded that reducing sodium intake and weight were feasible, effective, and safe lifestyle interventions for older persons with hypertension, recognizing participants were healthy. The 2023 ESH Guideline did not recommend weight loss for adults ≥ 80 years old unless obesity was severe, or the individual was robust given concerns of sarcopenia and malnutrition [13••].

#### Physical Activity 

A systematic review and meta-analysis assessed the effects of aerobic and resistance physical activity on the BP of adults ≥ 60 years [35]. Among more than 2200 individuals in the report, exercise lowered BP ~ 5.7/3.7 mmHg. Resistance exercise lowered BP roughly 0.7/0.7 mmHg more than aerobic activity. Moderate- and high-intensity aerobic exercise are recommended for BP reduction but may not be possible or preferred by many older adults. Low-intensity physical activity for six minutes hourly reduced systolic BP > 10 mmHg in overweight and obese highly sedentary adults (mean age 62 years) [36]. Other data suggest that low-intensity physical activity is similarly as effective as moderate- and high-intensity physical activity for diabetes prevention [37].

### Pharmacotherapy of Hypertension in Older Adults

The Hypertension Guidelines recommend the same classes of antihypertensive agents irrespective of age with preference for calcium channel blockers, thiazide-type diuretics, and renin-angiotensin system blockers, absent compelling indications for other drug classes [12, 13••]. The 2017 ACC/AHA Guideline is cautious in recommending initial single-pill combinations for older adults. The 2023 ESH Guideline cautiously recommends initial single-pill combinations for older adults with systolic BP 140–159 but is without reservation for older adults with systolic BP ≥ 160 mmHg.

#### Start Low and Go Slow Challenging the Status Quo

Guidance to start low and go slow is pervasive when initiating and intensifying antihypertensive pharmacotherapy for older adults with hypertension [11–13••]. In the U.S., antihypertensive medications are intensified on one in eight visits when BP is above target with an average follow-up interval of over 3 months [37]. At this rate, two years or more may be required to intensity pharmacotherapy for uncontrolled hypertension. In fact, most patients initiated on antihypertensive monotherapy remain on monotherapy 3 years later [38]. Initiating treatment with combination therapy rather than monotherapy is associated with better hypertension control at 6 months and one year as well as fewer CVDE [39–41]. Advising clinicians to go slow for older patients is not wise, especially when considering that CVDE are reduced more when BP is controlled during the first six months of treatment than time intervals compared to longer time intervals [42, 43].

To facilitate timely hypertension control, three sample treatment regimens are provided. The first regimen uses standard or half-maximal doses of a thiazide-type diuretic, angiotensin receptor blockers (ARB) and dihyrdropyridine calcium channel blocker (dCCB) added sequentially at monthly intervals, each of which would lower systolic BP ~ 9 mmHg or 27 mmHg total [44]. ARB and dCCB doses are doubled on the next monthly visit if BP is uncontrolled, which should lower systolic BP 4–6 mmHg. Chlorthalidone is doubled to the maximum recommended dose of 25 mg at month 5 for an additional 3–5 mmHg reduction in systolic BP or roughly 35–40 mmHg total. Hydrochlorothiazide at 25 and 50 mg, and indapamide 1.25 and 2.5 mg are roughly equally as effective to chlorthalidone 12.5 and 25 mg, respectively.

Following the same principles, the second regimen would lower systolic BP ~ 43 mmHg within 6 months with four antihypertensive drug classes, while the third regimen would lower systolic BP ~ 33 mmHg with three drug classes.

After initiating antihypertensive treatment, monthly follow-up visits with intensification of antihypertensive pharmacotherapy when BP is uncontrolled will increase the probability of controlling hypertension within 6 months [12]. Clinical judgment is required to determine if intensification of pharmacotherapy should be accelerated or delayed based on factors including distance from target BP, absolute risk for CVDE, and risk or occurrence of adverse effects.

### Periodic Reassessment of Comorbid Conditions, Physical and Cognitive Status, and Hypertension Target

Chronological aging is identical across individuals, whereas physiological aging is highly heterogeneous. Even within individuals, physiological aging is often non-linear. Comorbid conditions increase strongly as a function of age including dementia, frailty, coronary heart disease, congestive heart failure, stroke, and chronic kidney disease. Thus, periodic and comprehensive reassessment is warranted with annual assessment appropriate for many older individuals. More frequent assessment is appropriate when major, life-changing events occur, often marked by hospital or emergency department admission. That said, many patients with cancer are living longer and dying of CVDE [45]. Thus, some caution is appropriate in translating adverse health events as indications for less intensive management of hypertension and other CVD risk factors. A patient-centered approach, based on the best available data, is essential during the initial evaluation and selection of blood pressure target and treatment plan as well as appropriate revisions following changes in health status.

### An Important Role for Clinicians in Promoting Healthy Aging

Recent evidence suggests that antihypertensive treatment in midlife essentially eliminates the excessive age-related risk of cognitive decline [46]. Statin therapy, as noted earlier, reduces age-related arterial stiffening. Moreover, healthy lifestyle patterns including physical activity contribute to healthy aging. Of concern, following a period of declining disability in older U.S. adults toward the end of the last millennium, the more recent cohort of older adults shows evidence of increasing levels of disability [47]. Thus, it is important for clinicians to identify and control cardiovascular risk factors and to facilitate healthy lifestyle patterns earlier in life to promote healthy aging. Maintaining cognitive function and preventing or delaying moderate and severe frailty, which are key to retaining the benefits of more intensive therapy on CVDE. Success in this arena is vital to mitigating the projected burden of CVDE in a rapidly aging global population. Table 3 is provided to enhance the clinician’s effectiveness in managing older adults with hypertension.

**Table 3:** Key Clinical Points on Hypertension Management in Older Adults

1. As the global population ages, most clinicians will be seeing significantly more older adults with hypertension in their practices.
2. The benefits of intensive antihypertensive therapy outweigh risks in older adults with good cognitive function and absent moderate and severe frailty.
3. Hypertension in older adults is a women's health equity issue as women live longer than men and prevalent hypertension increases more and control falls more with age women.
4. Comprehensive assessment of cognitive and physical function is important in determining treatment intensity in a shared decision in older adults with hypertension. Periodic reassessment thereafter will inform whether changes in treatment intensity are warranted
5. In healthy older adults with hypertension, both sodium reduction and weight reduction are successful lifestyle interventions for improved BP control.
6. The adage "start low and go slow" contributes to clinical inertia in managing BP among older adults. Monthly reviews with treatment intensification if BP remains controlled are appropriate absent mitigating factors such as significant orthostatic hypotension and frailty.
7. Clinicians can play an important role in promoting healthy aging in younger and middle age adults to reduce cardiovascular risk and preserve benefits of intensive treatment at older ages.

### Supplementary Information

Below is the link to the electronic supplementary material.

Supplementary file1 (PDF 904 KB)

## Data Availability

All data in this manuscript are publicly available.
