# Supplementary material for: Managing Hypertension in Older Adults
Source: Curr Hypertens Rep. 2023 Dec 27;26(4):157–67. doi: 10.1007/s11906-023-01289-7 (PMC10904451; doi:10.1007/s11906-023-01289-7)
Supplement: Supplementary file 1 — Supplementary file1 (PDF 904 KB) [file 11906_2023_1289_MOESM1_ESM.pdf]

DALHOUSIE UNIVERSITY

GERIATRIC MEDICINE RESEARCH

FOR OFFICE USE ONLY
PF#: 20230901-04

PERMISSION TO USE THE CLINICAL FRAILTY SCALE (CFS)

The undersigned is granted permission to use, reproduce and distribute the Clinical Frailty Scale (CFS), developed by Dr. Kenneth Rockwood, in the format attached[1] for educational purposes and for non-commercially funded research and/or quality assurance projects. The CFS must be administered free of charge to patients and/or study participants. A formal Licensing Agreement is required for research funded by any commercial entity or pharma and, in some cases, for use in routine clinical care. The copyright holder reserves the right to prospectively follow-up at any time to determine whether use of the CFS meets the conditions described above. Reselling of the CFS or other commercial development without a license agreement is prohibited by copyright. The undersigned, their delegates and affiliated organization(s) agree that they will not claim ownership rights to the CFS, or any derivative, including translations, compilation, sequel or series. Nothing in this Agreement shall give the undersigned any right, title, or interest in the CFS other than the right to use in accordance with this Agreement. The CFS will not be modified unless explicit permission is granted.

**USER INFORMATION:**	
Full Name:	Brent M. Egan, MD
Position/Title:	Vice-President Cardiovascular Health Promotion
Institution/Organization:	American Medical Association
Mailing Address:	2 West Washington Street, Suite 601
Telephone:	864-474-7248
Email:	brent.egan@ama-assn.org
Type of organization:	☐ For-profit ☒ Not-for-profit ☐ Other, *please specify:*

INTENDED USE (Select all that apply):

☒ Reprint *Provide publication details:* *See note below

☐ Research study/clinical trial *Expected duration of study:* Start date End date
Describe use in study:
Is research sponsored or funded by pharma or industry? ☐ Y ☐ N
***If yes**, please specify:*

☐ Routine clinical care
Will the CFS be incorporated into an electronic medical/health record (EMR)? ☐ Y ☐ N
***If yes**, please identify whether an EMR company is involved (e.g., Epic, Meditech):*

☒ Other *Specify:* Review article for Current Hypertension Reports

Are you planning to translate the CFS? ☐ Y ☒ N ***If yes**, specify language(s):*
We request editable (e.g. MSWord) copies of all translations. We do not independently verify or validate translations.

Are you planning any commercial development that would incorporate the CFS?
☐ Y ☒ N ***If yes**, please specify:*

By your signature below, you attest that you understand the conditions under which permission is granted.

Signature:	*[signature]*
Date:	14 Aug 2023

Send completed, signed form by fax or email to:	**Geriatric Medicine Research**<br>1421-5955 Veterans' Memorial Lane, Halifax, NS B3H 2E1 Canada<br>Fax: 1-902-473-1050 | Email: gmru@dal.ca

Approved by: *[signature]* Date: September 1, 2023
Developer or Designated Authority

[1]A copy of the CFS will be sent to the user upon review and approval of this permission form. Valid only when signed by all parties.

*Additional details
The title of the review article is, 'Managing hypertension in older adults'.
The authors are Brent M. Egan, Holly J Mattaix-Kramer, Jan N. Basile, Susan E. Sutherland.
The journal is: Current Hypertension Report
Publisher: Springer
